# Antibiotics as Major Disruptors of Gut Microbiota

**DOI:** 10.3389/fcimb.2020.572912

**Published:** 2020-11-24

**Authors:** Jaime Ramirez, Francisco Guarner, Luis Bustos Fernandez, Aldo Maruy, Vera Lucia Sdepanian, Henry Cohen

**Affiliations:** ^1^ Gastroenterology and Nutrition Department, Instituto Nacional de Pediatria, Mexico City, Mexico; ^2^ Facultad Nacional de Medicina, Universidad Nacional Autonoma de Mexico, Mexico City, Mexico; ^3^ Digestive System Research Unit, Vall d’Hebron Institute of Research (VHIR), Barcelona, Spain; ^4^ Instituto de Gastroenterologia, Centro Medico Bustos Fernandez (CMBF), Buenos Aires, Argentina; ^5^ Catedra de Pediatria, Hospital Cayetano Heredia, Universidad Peruana Cayetano Heredia, Lima, Peru; ^6^ Division of Pediatric Gastroenterology, Pediatric Department, Escola Paulista de Medicina, Universidade Federal de São Paulo, São Paulo, Brazil; ^7^ Gastroenterology, National School of Medicine, Montevideo, Uruguay

**Keywords:** antibiotic overuse, antibiotic resistance, gut microbiota, microbial diversity, antibiotic-associated diarrhea (AAD), enterotypes

## Abstract

Advances in culture-independent research techniques have led to an increased understanding of the gut microbiota and the role it plays in health and disease. The intestine is populated by a complex microbial community that is organized around a network of metabolic interdependencies. It is now understood that the gut microbiota is vital for normal development and functioning of the human body, especially for the priming and maturation of the adaptive immune system. Antibiotic use can have several negative effects on the gut microbiota, including reduced species diversity, altered metabolic activity, and the selection of antibiotic-resistant organisms, which in turn can lead to antibiotic-associated diarrhea and recurrent *Clostridioides difficile* infections. There is also evidence that early childhood exposure to antibiotics can lead to several gastrointestinal, immunologic, and neurocognitive conditions. The increase in the use of antibiotics in recent years suggests that these problems are likely to become more acute or more prevalent in the future. Continued research into the structure and function of the gut microbiota is required to address this challenge.

## Introduction

Scientific advances made in recent decades have led to an increased recognition of the role of the human gut microbiota in health and disease (Ananthakrishnan et al., 2019). Prior to that, little research had been conducted into the non-pathogenic microorganisms that inhabit the gastrointestinal tract (Guarner, 2012). Because most of these microorganisms cannot be cultured, they remained largely unexplored before the advent of molecular techniques. Ample experimental and clinical evidence now shows that gut microorganisms are required for the optimal functioning of the human body (Guarner, 2015).

In 1885, Louis Pasteur hypothesized that animals raised in sterile conditions would not be able to survive (Pasteur, 1885). Bernard S. Wostmann and his team proved Pasteur’s hypothesis wrong when they developed methods for breeding animals in germ-free conditions (Wostmann, 1981). However, they discovered that germ-free animals required large quantities of nutrient-rich food, yet continued to have stunted growth and development compared with normal animals. Germ-free animals had smaller hearts, lungs, and livers, lower cardiac output, thinner intestinal walls, reduced gastrointestinal motility, lower serum gamma globulin levels, and atrophied lymph nodes (Wostmann, 1981). Most of these deficiencies can be restored by introducing intestinal microbiota from animals raised under normal conditions. Therefore, while microbial colonization may not be essential for life, it is critical for health (Guarner and Malagelada, 2003; O’Hara and Shanahan, 2006).

The knowledge gained from germ-free animal models is currently being translated to human physiology and medicine through research into commensal microorganisms (Gilbert et al., 2018). Ecological niches located along the gastrointestinal tract, from mouth to anus, contain the largest microbial communities found in the human body, comprising approximately 3.9×10^13^ bacterial cells, which can be investigated in fecal samples using a combination of genomic and culture-based approaches (Browne et al., 2016; Sender et al., 2016). Magnetic resonance imaging indicates that the large intestine is inhabited by several hundred grams of microbes, which is not surprising because it has the optimal conditions for microbial proliferation (i.e., constant temperature, anaerobiosis, and slow motility) (Bendezú García et al., 2016).

The development of antibiotics is widely regarded as one of the greatest medical advances of the 20^th^ century (Bud, 2007; Royal College of Physicians of Edinburgh, 2010). Worldwide antibiotic use increased by 65% between 2000 and 2015 (Klein et al., 2018). Although most courses of antibiotic treatment have no apparent adverse effects, antibiotics can cause significant changes in gut microbiota that have both short- and long-term health consequences (Dethlefsen and Relman, 2011). An association between early antibiotic exposure and childhood asthma, allergies, and airway illnesses has long been suspected (Foliaki et al., 2009). Observational studies also implicate antibiotic use in the pathogenesis of other increasingly prevalent conditions, including gastrointestinal infections, weight gain and obesity, inflammatory bowel disease (IBD), and colorectal cancer (Lange et al., 2016). Another serious consequence of antibiotic use is the development of bacterial antibiotic resistance (Giedraitiene et al., 2011).

The aim of this article is to review the literature on the gut microbiota and its importance in human health, as well as to describe the risks associated with the use of antibiotics and to outline some of the approaches that can minimize these risks.

## Human Gut Microbiota

Microbiota is defined as the assemblage of microorganisms present in a particular environment, while the term “microbiome” refers to the entire habitat, including the microorganisms (bacteria, archaea, lower and higher eurkaryotes, and viruses), their genomes and the environmental conditions that obtain in that habitat (Marchesi and Ravel, 2015). Bacteria, archaea, eukaryotes (fungi and protists), and viruses that inhabit the gastrointestinal tract of humans are collectively referred to as the human gut microbiota.

### Composition

The genome of a single symbiotic microbial species is likely to be insufficient for survival (Guarner, 2015). As a result, multispecies communities organized around a complex network of metabolic interdependencies, such as those found in the human gut, represent the natural environment for most symbiotic microbes (Guarner, 2015).

The development of culture-independent research methods that combine genetic sequencing with bioinformatics has led to rapid advances in the study of the human gut microbiota (Guarner, 2015). One of the most common methods used for taxonomic identification and assessment of species diversity of prokaryotes (Bacteria and Archaea) is sequencing of the gene that encodes the small subunit of ribosomal RNA (16S rRNA) (Guarner, 2015). Of the 55 phyla that comprise the domain Bacteria, only seven to nine are found in the human gut, with the majority (90%) belonging to the *Bacteroidetes* and *Firmicutes* phyla (Eckburg et al., 2005). Other phyla consistently identified in the human gut include *Proteobacteria*, *Actinobacteria*, *Fusobacteria*, and *Verrucomicrobia*, while very few Archaea species have been detected (Eckburg et al., 2005).

Another culture-independent research method is whole genome sequencing, which yields an inventory of all the genes present in a sample. Whole genome sequencing also allows the analysis of the functional and metabolic networks, as well as the detection of genes that belong to non-bacterial members of the microbial community, such as viruses, yeasts, and protists. In total, approximately 10 million non-redundant microbial genes have been identified in human fecal samples (Li et al., 2014). On average, 600,000 non-redundant microbial genes are present in the gastrointestinal tract of a human, of which 300,000 genes are shared by people living in Europe, North America, and China (Li et al., 2014).

There are differences between the microbial communities inhabiting the lumen and mucosa within the same individual (Eckburg et al., 2005; Donaldson et al., 2016). Furthermore, bacterial species found in the lumen vary from the cecum to the rectum. In the cecum, slow transit time and lack of simple sugars promote the proliferation of fermentative polysaccharide-degrading anaerobes, notably *Prevotella*, *Roseburia*, *Faecalibacterium*, *Lachnospira*, and *Eubacterium*. In distal sections of the colon, mucolytic and proteolytic species are common (e.g., *Bacteroides*, *Ruminococcus*, *Akkermansia*, *Bifidobacterium*, *Methanobrevibacter*, *Desulfovibrio*, *Proteus*, and *Escherichia*). Mucosa-associated bacteria from the terminal ileum to the rectum tend to be more stable at the phylum and genus level, but patches of heterogeneity within the same intestinal area have been described (Donaldson et al., 2016).

Most strains that comprise the gut microbiota are resident for decades, although their relative abundance varies over time in a given individual (Donaldson et al., 2016). However, longitudinal studies show that factors such as diet, drug intake, lifestyle (smoking, travelling, physical activity), co-morbidities, or colonic transit time have an impact on the microbial composition of fecal samples obtained from a unique host (Gilbert et al., 2018; Allaband et al., 2019; Zmora et al., 2019). Although intra-individual changes in gut microbiota composition can be significant, for example, due to an episode of acute infectious diarrhea or after antibiotic treatment, over time it tends to return to its pre-disturbance state, a quality known as resilience (Gilbert et al., 2018; Allaband et al., 2019). The diversity of gut microbiota also changes with age, increasing from infancy to adulthood and decreasing in the elderly. Gut microbial changes in elderly individuals correlate with measures of frailty, nutritional status, and markers of inflammation, suggesting that diet-driven microbiota alterations play a role in the varying rates of age-associated health decline (Kuipers, 2019).

The gut microbiota of each individual person contains many unique strains not found in other individuals, and inter-individual differences in microbiota composition are much larger than intra-individual variations (Allaband et al., 2019). Sex, ethnicity and geographic location affect the taxonomic composition of the microbiome (Gaulke and Sharpton, 2018). For example, the fecal microbiota of adults from the metropolitan areas of Europe and North America is less diverse compared with adults from rural populations in Africa and South America (Clemente et al., 2015; Sonnenburg and Sonnenburg, 2019).

### Enterotypes

Despite intra- and inter-individual differences, an analysis of the microbial composition of fecal samples from American, European, and Japanese individuals showed similarities in the structure of gut microbiota at the genus level (Arumugam et al., 2011). Multidimensional scaling and principal coordinates analysis revealed the existence of three clusters, or enterotypes, each of which was characterized by the predominance of one genus: *Bacteroides* (enterotype 1), *Prevotella* (enterotype 2), or *Ruminococcus* (enterotype 3). Clustering was not driven by age, sex, nationality, or body mass index (Arumugam et al., 2011). The results of this study indicate that there are a limited number of well-balanced, host-microbiota symbiotic states. In addition, the discrete nature of these states suggests that the structure of the human gut microbiota is primarily determined by interactions between various bacterial genera (Guarner, 2015).

The clinical implications of enterotypes have been the subject of several studies (Costea et al., 2018). The *Bacteroides* enterotype has been associated with decreased microbial genetic diversity, insulin resistance, and the risk of obesity and non-alcoholic steatohepatitis (Le Chatelier et al., 2013; Costea et al., 2018). Perhaps unsurprisingly, long-term dietary patterns may be one of the factors that determine the enterotype (Wu et al., 2011). Diets rich in animal protein and fat are associated with the *Bacteroides* enterotype, while diets characterized by the predominance of plant carbohydrates are associated with the *Prevotella* enterotype (Wu et al., 2011).

### Functions

**Figure 1:**
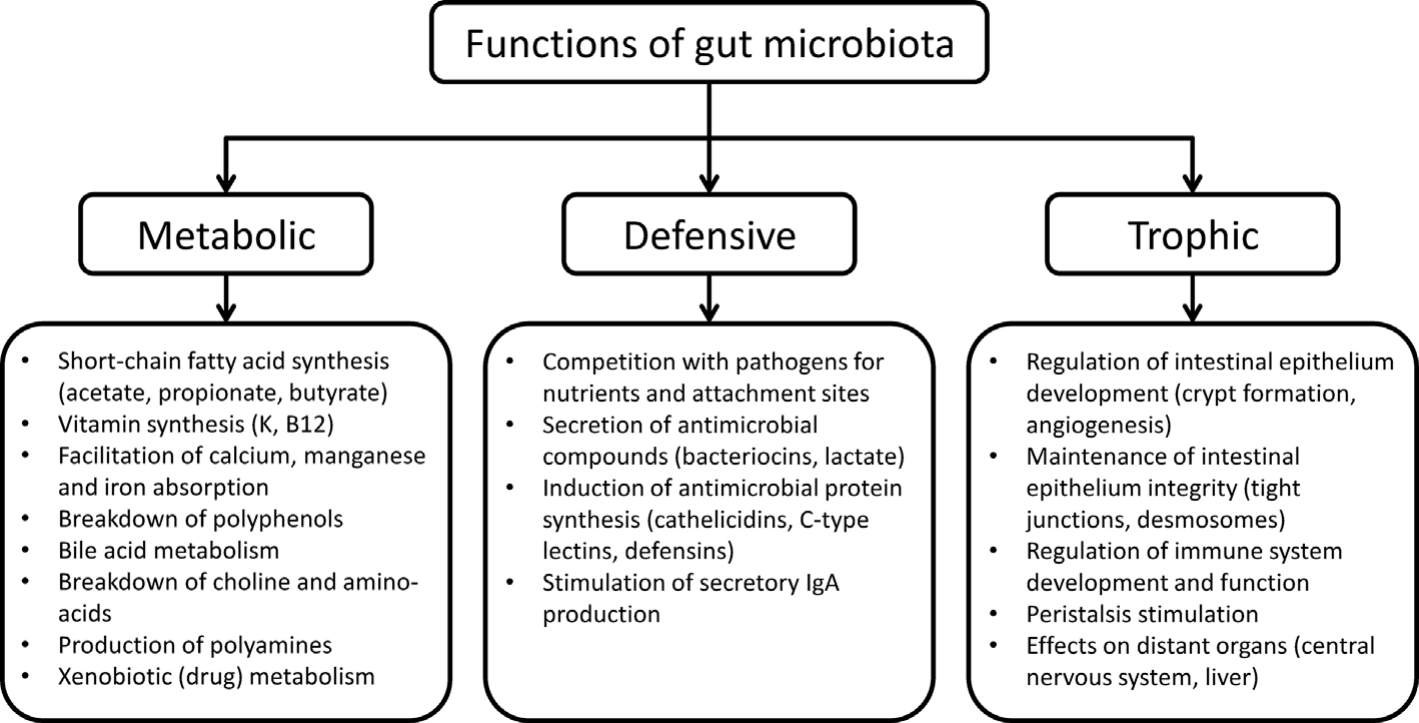
Functions of human gut microbiota.

The functions of the human gut microbiota fall into three categories, namely metabolic, defensive, and trophic functions (Guarner and Malagelada, 2003) (**Figure 1**).

The metabolic function involves the fermentation of non-digestible dietary substrates and recovery of energy and nutrients. In humans, digestion of vegetables, fruits, nuts, and wholegrain cereals is mostly performed by gut microbes since our enzymatic resources for the digestion of carbohydrates are limited to amylases and disaccharidases. In addition, fermentation of complex carbohydrates in the colon produces short-chain fatty acids that are absorbed by the host (Litvak et al., 2018). Butyrate produced by *Faecalibacterium prausnitzii* and others inhibits interleukin-17, generates regulatory T cells and has anti-inflammatory effects in experimental models (Litvak et al., 2018). Colonic microorganisms also break down xenobiotics and other foreign compounds, contribute to amino acid and vitamin synthesis, and provide a variety of nutrients in individuals on monotonous diets (Zmora et al., 2019).

The gut microbiota performs a defensive function by competing for attachment sites and nutrients with indigenous fungi or bacteria with pathogenic potential, such as *Candida* and *Clostridioides difficile*, thereby preventing invasion or overgrowth of these organisms (McFarland, 2008). Resident microorganisms also inhibit the growth of their competitors by producing bacteriocins (McFarland, 2008).

The trophic function of gut microbiota includes promotion of epithelial cell proliferation and differentiation, stimulation of intestinal motor activity and neuro-endocrine pathways of gut origin, and regulation of the immune system and the central nervous system (Mayer et al., 2015).

Induction and regulation of the adaptive immune system is one of the major aspects of the trophic function of the gut microbiota. The large surface area of the gastrointestinal tract is constantly exposed to a variety of antigens. As a result, intestinal immunity is the largest and most complex part of the overall immune system, with at least 80% of all antibodies produced in adults originating in the intestinal mucosa (Brandtzaeg, 2010). The intestinal immune system must be able to discriminate between pathogens and antigens derived from food or from commensal non-pathogenic microbes, because an inflammatory reaction to a foreign antigen can also harm the host (Tanoue et al., 2016). The gut microbiota affects the development of the adaptive immune system by stimulating the growth of lymphoid structures, T- and B-cell differentiation and the establishment of immune tolerance (Zhao and Elson, 2018).

### Perturbation of the Gut Microbiota

The term dysbiosis refers to a persistent perturbation of the gut microbiota, and has been defined as an alteration in both the composition and function of the microbiota caused by host-related and environmental factors that overwhelms the resistance and resilience capabilities of the microbial ecosystem (Levy et al., 2017). Alterations in gut microbiota may be implicated in the pathogenesis of several non-communicable diseases and in the transition of these conditions to chronicity. Numerous studies have shown links between changes in the composition of the gut microbiota and diseases, including recurrent diarrhea associated with *C. difficile*, some bowel disorders (including IBD), colorectal cancer, non-alcoholic steatohepatitis, type 2 diabetes, obesity, and advanced chronic liver disease (Duvallet et al., 2017; Wirbel et al., 2019). However, for some of these examples, the studies are inconsistent, possibly because the methodology has not been standardized. Furthermore, rather than necessarily indicating a causative role in the pathogenesis of a disease, these associated microbiota changes could be a consequence of the disease itself. Thus, follow-up cohort studies are needed, particularly studies of interventions that may restore the composition of gut microbiota.

Nevertheless, studies in rodents have shown that it is possible to use fecal transplants to transfer certain disease phenotypes, including insulin resistance, obesity, anxiety, and intestinal inflammation. This suggests that some gut microbiota changes may have a causative role in those diseases. In humans, fecal microbiota transplantation has a well-established role in the treatment of recurrent diarrhea caused by *C. difficile* infection (CDI) (Cammarota et al., 2017). Fecal transplant has become standard therapy for this condition as recommended by national guidelines (Mullish et al., 2018). Less successful results have been observed with attempts to treat IBD. There are four randomized trials that evaluated fecal transplant as induction therapy for achieving remission in active ulcerative colitis, and these collectively show statistical improvement compared with control. At 8 weeks, 37% of participants in the stool transplant group were in remission compared with 18% of participants in the control group (Imdad et al., 2018). Additional studies are needed to further define transplant material rich in microbial populations identified as missing in the patient, and avoiding transplants rich in aggressive, over-represented microbes.

A loss of species diversity seems to be a common feature of a disturbed gut microbiota. Low microbial richness is associated with an overabundance of pro- versus anti-inflammatory microbial species, and this may lead to intestinal inflammation and disrupted function of the mucosal barrier. In a study that used the number of microbial genes in fecal samples as a proxy for diversity, individuals with low gene counts were more likely to have insulin or leptin resistance, adiposity, or dyslipidemia, and a more marked inflammatory phenotype, compared with those with high gene counts (Le Chatelier et al., 2013). This association might indicate that low microbial richness increases an individual’s risk of developing metabolic syndrome (i.e., obesity, arterial hypertension, type 2 diabetes, dyslipidemia, and non-alcoholic steatohepatitis).

In terms of the functional capacity of the gut microbiota, a low microbial gene count appears to be associated with a reduced production of short-chain fatty acids (particularly butyrate) for the host (Le Chatelier et al., 2013). Failure to produce butyrate increases the flow of oxygen towards the mucosa and perturbs the micro-ecosystem in a way that favors the survival of oxygen-resistant bacteria and precludes recovery of strict anaerobes (Litvak et al., 2018). In this way, dysbiosis has been described as disruption of the symbiotic balance between microbiota and host. Such changes critically affect the resilience capacity of the ecosystem and increase the likelihood that the imbalance will become chronic.

## Effects of Antibiotics on Gut Microbiota

Antibiotic treatment reduces the overall diversity of gut microbiota species, including loss of some important taxa, which causes metabolic shifts, increases gut susceptibility to colonization, and stimulates the development of bacterial antibiotic resistance (Lange et al., 2016).

### Reduced Diversity

**Figure 2:**
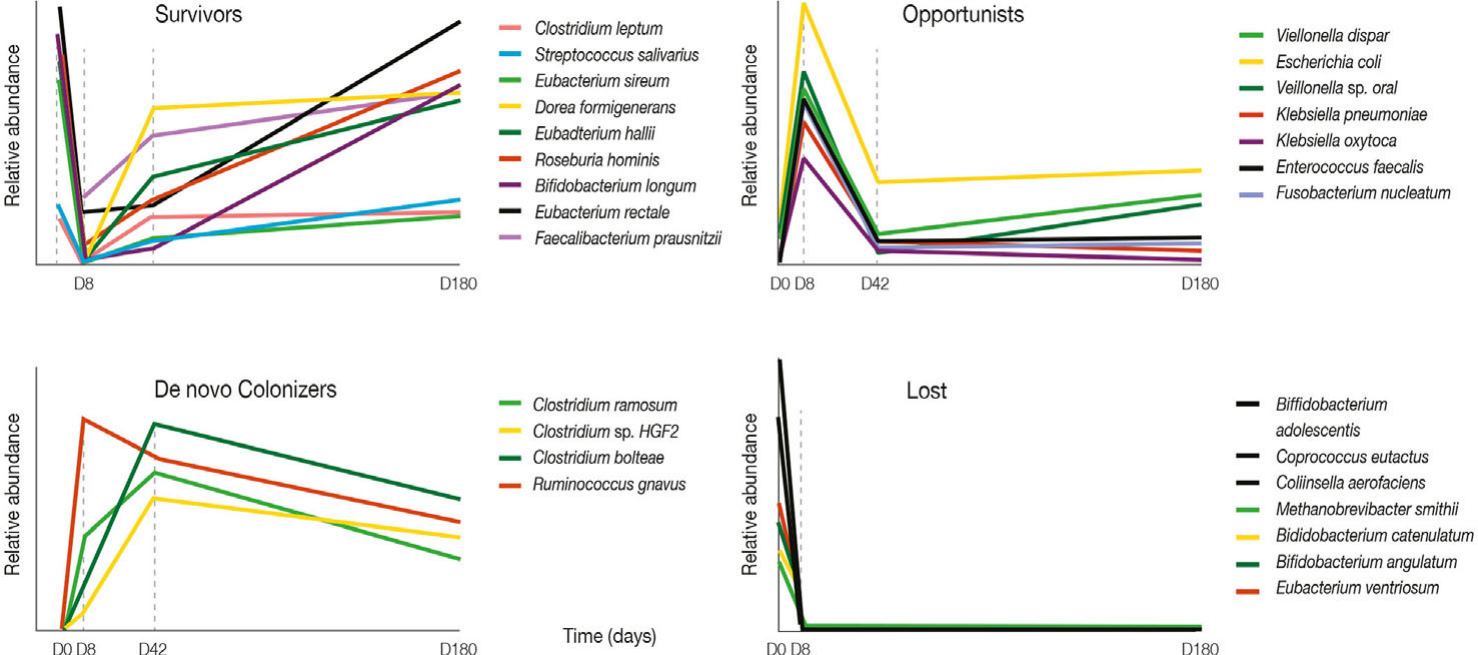
Four-day antibiotic treatment induced large shifts in bacterial abundances. Graphs show relative abundance of representative species according to their pattern of change after four-day antibiotic treatment over a 180-day follow-up period. Adapted from Palleja et al. (2018).

Antibiotic use is associated with reduced microbiota diversity. In children, restoration of microbial diversity following antibiotic treatment has been reported to take approximately 1 month (Yassour et al., 2016). In adults, administration of a combination of meropenem, gentamicin, and vancomycin resulted in an increase in the prevalence of *Enterobacteriaceae* and other pathobionts, and a decrease in *Bifidobacterium* and butyrate-producing species (Palleja et al., 2018) (**Figure 2**). While the baseline composition of the gut microbiota was mostly restored within 1.5 months, several common species remained undetectable for the rest of the observation period (180 days) (Palleja et al., 2018).

Antibiotics can also disrupt the balance that normally exists between the various species of gut microbiota. For instance, by causing a decrease in species diversity, antibiotics can lead to the overgrowth of pathobionts, such as toxigenic *C. difficile* (Ianiro et al., 2020).

It should be noted that reduced diversity does not necessarily mean a reduced number of bacteria overall. As the antibiotic-susceptible bacteria are eliminated, antibiotic-resistant bacteria multiply and take their place. In fact, the total microbial load may increase after antibiotic treatment, even though species diversity is reduced. In a study of patients treated with broad-spectrum antibiotics, treatment with β-lactams over the course of 7 days increased the microbial load in fecal samples two-fold (Panda et al., 2014). In this study, broad-spectrum antibiotic use also increased the ratio of *Bacteroidetes* to *Firmicutes* (Panda et al., 2014).

### Altered Metabolome

The complete set of small molecules (<1500 Da) found in a biological system is referred to as the metabolome of that system (Lamichhane et al., 2018). The effects of antibiotics on the gut metabolome are less well studied than their effects on gut microbial diversity. One factor that makes research into this connection more complex is metabolomic redundancy. Despite these challenges, some of the effects of antibiotics on the gut metabolome in mice have been described (Cho et al., 2012; Choo et al., 2017). In a study in young mice, low-dose antibiotics led to increased adiposity and elevated hormones associated with the metabolism of carbohydrates, lipids and cholesterol (Cho et al., 2012). In another study, vancomycin-imipenem administration resulted in increased levels of arabinitol and sugars (e.g. sucrose) in feces (Choo et al., 2017). Elevated levels of these compounds have been associated with an increased susceptibility to CDI, potentially by serving as a growth substrate. Vancomycin/imipenem also reduced the relative abundance of *Lachnospiraceae* and *Ruminococcaceae* bacteria that normally convert arabinitol to pentose sugars. Immediately after the cessation of vancomycin/imipenem, a small but significant reduction in the level of arginine was observed that correlated with increased prevalence of *Escherichia* and *Shigella* species and reduced prevalence of *Ruminococcaceae* and *Bacteroides*. An increase in arginine observed 9 days after the end of vancomycin/imipenem was correlated with increased prevalence of organisms from the *Enterobacter* genus and reduced prevalence of *Alistipes*. Arginine serves as a precursor for a number of immunomodulatory compounds (Choo et al., 2017).

Antibiotic exposure produces changes in the gut metabolome that may or may not correlate with changes in the microbiome. In patients with metabolic syndrome, oral administration of vancomycin decreased fecal secondary bile acids, with a simultaneous postprandial increase in primary bile acids in plasma. Concomitantly, vancomycin affected host physiology by decreasing peripheral insulin sensitivity (Vrieze et al., 2014). Antibiotic-induced changes in bile acid metabolism may affect host physiology and susceptibility to infection.

### Antibiotic Resistance

Antibiotic resistance is defined as the capacity of a species of bacteria to survive antibiotic concentrations that inhibit or kill other bacteria of the same species (Sabtu et al., 2015). It first evolved in antibiotic-producing bacteria as a way to defend against their own products and compete with other microbes (Rolain, 2013). Worldwide, antibiotic resistance has emerged as a significant public health concern. Between 2000 and 2015, global antibiotic consumption increased by 65% (Klein et al., 2018), with amoxicillin and amoxicillin/clavulanic acid being the most commonly used antibiotics (World Health Organization, 2018). Between 2000 and 2015, the greatest increases in antibiotic use were observed in developing countries and the gap between developing and developed countries decreased. Antibiotic resistance is responsible for an estimated 35,000 deaths in the US and 25,000 deaths in Europe each year (European Medicines Agency and European Centre for Disease Prevention and Control, 2009; Centers for Disease Control and Prevention, 2019). According to one estimate, by 2050 the number of deaths per year caused by antibiotic resistance will be 317,000 in North America, 390,000 in Europe, 392,000 in Latin America, 4,150,000 in Africa, and 4,730,000 in Asia (Sugden et al., 2016). The World Health Organization estimates that the number of antibiotic resistance-related deaths could reach 10 million by 2050 (World Health Organization, 2019). In China, resistance has increased dramatically due to large-scale antibiotic use in livestock (Van Boeckel et al., 2015). Inappropriate use of antibiotics, facilitated by their availability over the counter and on the internet, is the primary driver of antibiotic resistance (Sabtu et al., 2015). The increased prevalence of antibiotic-resistant bacteria results in infections that are difficult and expensive to treat. As older antibiotics lose effectiveness due to resistance, newer antibiotics must be used. However, these drugs are more expensive and may not be available to many of those who need them, particularly in countries with a high burden of infectious diseases (Klein et al., 2018).

Bacteria have developed a range of processes to elude the effects of antibiotics, including protection against the uptake of antibiotics through their cell membranes, developing enzymatic processes that modify or degrade the antibiotic, altering the molecules that antibiotics target, and actively removing antibiotics from the cell *via* specialized efflux proteins (Giedraitiene et al., 2011). Bacterial enzymes that are able to neutralize antibiotics include β-lactamases, aminoglycoside-modifying enzymes, and chloramphenicol acetyltransferases (Giedraitiene et al., 2011). Bacteria are also able to mutate the molecular targets of antibiotics, interfering with the highly specific interaction between the antibiotic and its target molecule through small structural modifications. For example, mutations in penicillin-binding proteins reduce the efficacy of β-lactams, mutations in 23S rRNA confer resistance to macrolides, lincosamides and streptogramin B, and mutations in DNA topoisomerase II and IV lead to resistance to quinolones and fluoroquinolones (Giedraitiene et al., 2011). Bacteria can eliminate antimicrobial agents by pumping them out *via* efflux proteins located in the bacterial cell membrane. Although these proteins can be antibiotic-specific, most are multidrug transporters. Another mechanism of resistance is reduced outer membrane permeability, which results in a decreased uptake of antibiotics (Giedraitiene et al., 2011).

In humans, the gut microbiota contains a pool of antibiotic resistance genes. Antibiotic treatment rapidly increases the pool of resistance genes present in the gut, which slowly declines after treatment is discontinued (Rolain, 2013). Antibiotic-resistant gut bacteria can be transferred to a newborn from his/her mother at birth and thereafter may persist for weeks. In a Swedish study, tetracycline resistance was detected in 12% of commensal *E. coli* strains from infants who had not received tetracycline (Karami et al., 2006).

## Clinical Consequences of Antibiotic Use

### Short- and Medium-Term Consequences

#### Antibiotic-Associated Diarrhea

Antibiotic-associated diarrhea (AAD) is defined as diarrhea that occurs in association with the administration of antibiotics and that cannot be explained otherwise (Bartlett, 2002). Diarrhea can occur during antibiotic treatment and up to 8 weeks after treatment cessation (McFarland, 2008).

Under normal conditions, homeostasis of the intestinal epithelium is maintained by a number of mechanisms, including a thick mucus layer and tight junctions to maintain the integrity of the intestinal epithelium (Willing et al., 2011). The number of gut bacteria is controlled by antimicrobial peptides (C-type lectins, defensins, and cathelicidins), which are secreted into the mucus layer along with secretory immunoglobulin A (IgA) in response to microorganism-associated microbial patterns. Antibiotic exposure eliminates subsets of the normal gut microbes, thereby reducing exposure to microorganism-associated microbial patterns and decreasing secretion of antimicrobial peptides (Willing et al., 2011). In addition, some antibiotics cause thinning of the mucus layer and disruption of tight junctions, rendering the intestinal epithelium more susceptible to damage. Changes in microbial proteases could also affect mucosal barrier function. Collectively, these processes facilitate successful invasion by enteric pathogens (Willing et al., 2011).

The prevalence of AAD among patients who receive antibiotics is approximately 5–35% (McFarland, 2008). In a study of adult ambulatory patients receiving antibiotics for 5–10 days, the incidence of AAD was 17.5% (Beaugerie et al., 2003). The clinical course of AAD differed depending on whether *C. difficile* was involved, with most episodes of non-*C. difficile* AAD being mild in severity and self-limiting, lasting only a few days (Beaugerie et al., 2003).

Meta-analyses have indicated that specific strains of probiotics may be useful for the prevention of AAD. Compared with placebo or no treatment, the risk of AAD among adults and children treated with antibiotics was significantly reduced with probiotic therapy comprised of *Lactobacillus rhamnosus* GG (relative risk, 0.49; 95% CI, 0.29–0.83) (Szajewska and Kolodziej, 2015a) or *Saccharomyces boulardii* (risk ratio, 0.47; 95% CI, 0.38–0.57) (Szajewska and Kolodziej, 2015b).

The 2016 European Society for Paediatric Gastroenterology, Hepatology and Nutrition guidelines on probiotics and prebiotics recommend *L. rhamnosus* GG and *S. boulardii* for prevention of AAD in children, in both cases as a strong recommendation with moderate quality of evidence (Szajewska et al., 2016).

#### *C. difficile*-Associated Diarrhea

*C. difficile* is a Gram-positive, spore-forming, anaerobic bacillus with spores that are highly resistant to desiccation, chemicals, and extreme temperatures, and can remain viable for years (Edwards et al., 2016). *C. difficile* produces toxins A and B that can damage the gut mucosa (Kuehne et al., 2010).

In 2011, 453,000 cases of CDI and 29,000 CDI-associated deaths were recorded in the US (Leffler and Lamont, 2015). A significant proportion of CDIs are nosocomial, with the incidence of CDI being approximately 20 cases per 100,000 person-years in the community and approximately 15 per 1000 hospital discharges (Leffler and Lamont, 2015). The most significant risk factor for CDI is antibiotic use, with the most commonly associated drugs being ampicillin, amoxicillin, cephalosporins, clindamycin, and fluoroquinolones. Both the duration of antibiotic therapy and the number of drugs used are positively correlated with higher CDI risk. Other risk factors include old age, compromised immunity, and hospitalization, particularly in an intensive care unit (Leffler and Lamont, 2015).

Diagnosis of CDI in patients with diarrhea is based on the presence of bacteria in the stools. CDI is considered severe if serum albumin levels are <3 g/dl and either the white blood cell count is ≥15,000 per mm^3^ or abdominal tenderness is present (Surawicz et al., 2013). A two-step algorithm for detecting *C. difficile* in stool samples has been proposed that allows rapid diagnosis within 4 h. Step 1 consists of detecting *C. difficile*-specific glutamate dehydrogenase antigen and step 2 is detection of toxin A or B. Approximately 87.3% of suspected cases can be excluded at the first step. If no toxin is detected at the second step, culture should be performed (Fenner et al., 2008).

Recommended treatments for CDI include metronidazole 500 mg orally three times daily for 10 days in mild and moderate cases, and vancomycin 125 mg four times daily for 10 days in severe cases. Antibiotics that led to CDI should be discontinued if possible (Surawicz et al., 2013; Neut et al., 2017).

Several meta-analyses have evaluated the use of probiotics for prevention of CDI in children or adults receiving antibiotics. Two meta-analyses showed that the incidence of CDI is lower in adult patients who receive probiotics in addition to antibiotic treatment than in those who received placebo (Johnson et al., 2012; Goldenberg et al., 2013), while another meta-analysis produced similar findings in children (Goldenberg et al., 2015). Despite these findings, the evidence that probiotics prevent CDI was considered insufficient to support their routine use (Surawicz et al., 2013).

#### Helicobacter pylori Infection

*Helicobacter pylori* (Hp) is a Gram-negative, spiral-shaped, micro-aerophilic bacterium that colonizes the gastric mucosa of humans (Perez-Perez and Blaser, 1996; Suerbaum and Michetti, 2002). Hp infection produces an inflammatory response in the gastric mucosa. In most cases, this is a low-level and asymptomatic reaction. However, in some individuals, the infection can lead to gastric and duodenal ulcers, intestinal metaplasia, and gastric cancer (Suerbaum and Michetti, 2002).

Currently, in areas of high clarithromycin resistance (>15%), bismuth quadruple or non-bismuth quadruple concomitant (e.g., proton-pump inhibitor, amoxicillin, clarithromycin, and metronidazole) therapies are recommended (Malfertheiner et al., 2017). However, in areas of both high clarithromycin and metronidazole resistance, bismuth quadruple therapy is recommended as first-line therapy (Malfertheiner et al., 2017).

Hp eradication can have both positive and negative effects. On the one hand, eradication of Hp restores the microbiota composition in line with Hp-negative controls (Li et al., 2017). On the other hand, Hp eradication has been reported to cause changes in microbiota composition that can negatively affect the host (Yap et al., 2016). Hp eradication was associated with a decrease in the relative abundance of *Bacteroidetes* and an increase in *Firmicutes*. Increasing the prevalence of bacteria that produce short-chain fatty acids can lead to an increased risk of metabolic disorders (Yap et al., 2016). Failure of Hp eradication therapy has been linked to poor compliance due to antibiotic-associated adverse events (Losurdo et al., 2018).

An Hp eradication strategy that combined probiotics (*S. boulardii*) with triple therapy was more effective and had a better adverse event profile than placebo or no intervention plus triple therapy (Szajewska et al., 2010; Szajewska et al., 2015). The addition of *Lactobacillus*- and *Bifidobacterium*-containing probiotics to Hp eradication therapy was also associated with an improved effectiveness and safety profile relative to eradication therapy without probiotics (Wang et al., 2013; Losurdo et al., 2018). In a meta-analysis of studies in patients receiving antibiotic therapy for Hp eradication, *L. acidophilus*, *L. casei* DN-114001, *L. gasseri*, and *Bifidobacterium infantis* 2036 supplementation was associated with significantly higher eradication rates compared with controls (no probiotic supplementation) (Dang et al., 2014).

### Long-Term Consequences

Antibiotics are widely used in infants and children, systemic antibiotics being the most commonly prescribed drugs for children in the US (Chai et al., 2012). Antibiotic use in childhood has been associated with several negative outcomes later in life, including the development of obesity, asthma, allergy, and IBD (Cho et al., 2012; Bokulich et al., 2016; Korpela et al., 2016; Turta and Rautava, 2016).

Exposure to antibiotics during infancy has been associated with delayed gut microbiota development. In a study of infants aged ≤2 years, the delay in microbiota development after antibiotic use was particularly pronounced between the ages of 6 and 12 months (Bokulich et al., 2016). *Enterobacteriaceae*, *Lachnospiraceae*, and *Erysipelotrichaceae* species were among the operational taxonomic units that were depleted by antibiotics (Bokulich et al., 2016). Furthermore, frequent use of macrolide antibiotics in the first 2 years of life was significantly associated either with current asthma and obesity or with the subsequent development of these conditions (Korpela et al., 2016).

Early exposure to antibiotics and resulting dysbiosis are thought to contribute to IBD pathogenesis. A population-based cohort study showed that infants who received anti-anaerobic antibiotics in the first year of life were more likely to be diagnosed with IBD than those not treated with antibiotics (Kronman et al., 2012). Similarly, a nationwide Danish cohort study of children reported that the probability of developing IBD was highest in the first 3 months after antibiotic use and in children who had received at least seven courses of antibiotics (Hviid et al., 2011). A nationwide Finnish case-control study also showed a stronger association between Crohn’s disease and antibiotic use in boys than in girls; the antibiotic class with the strongest association with Crohn’s disease was cephalosporins (Virta et al., 2012).

Human genetics and diet are known to play an important role in determining body weight; however, it is now widely accepted that the increased prevalence of obesity over the past 30 years may also be attributable to alterations in gut microbiota composition (Biedermann and Rogler, 2015). In particular, early-life antibiotic exposure has been associated with the development of adiposity in humans (Trasande et al., 2013).

## Conclusions

Recent research has shown the gut microbiota to be an intricate network of metabolically interdependent microorganisms. A symbiotic gut microbiota performs several vital functions by aiding digestion, stimulating and regulating the immune system and preventing the growth of pathogens. The increasingly widespread use of antibiotics is a major cause for concern as antibiotics disrupt the gut microbiota. In order to address this challenge, further research is necessary to improve our understanding of the gut microbiota and strategies to reverse these disruptions, including the use of probiotics.

## Author Contributions

All authors (JR, LB, VS, FG, AM, and HC) wrote, edited, and reviewed the drafts of the manuscript, and agree to be accountable for the content of the work. All authors contributed to the article and approved the submitted version.

## Funding

The development of this review article was funded by Biocodex France. The funder was not involved in the study design, collection, analysis, interpretation of data, the writing of this article or the decision to submit it for publication.

## Conflict of Interest

FG is a member of the advisory boards at Biocodex and Instituto Danone; and has received research grants from AB Biotics, Takeda and Abbvie. AM is a speaker and member of the advisory board at Biocodex France. HC is an advisor for Biocodex and Megapharma.

The remaining authors declare that the research was conducted in the absence of any commercial or financial relationships that could be construed as a potential conflict of interest.
